# Assessment of Risk to the U.S. Population from the Ebola Disease Outbreak Caused by Bundibugyo Virus, 2026

**DOI:** 10.15585/mmwr.mm7522e2

**Published:** 2026-06-11

**Authors:** Danielle M. Richard, Isobel Routledge, Sarah Koeller, Isaac Ghinai, Christopher H. Hsu, Kevin Chatham-Stephens, Beau B. Bruce, Rebecca Kahn, Inga Holmdahl

**Affiliations:** ^1^Inform Division, Center for Forecasting and Outbreak Analytics, CDC; ^2^Goldbelt Ltd., Washington, DC; ^3^CDC 2026 Ebola Response; ^4^Predict Division, Center for Forecasting and Outbreak Analytics, CDC.

**Summary**

**What is already known about this topic?**

An outbreak of Bundibugyo virus disease (BVD), a type of Ebola disease, is currently occurring, centered in the Ituri province of the Democratic Republic of the Congo (DRC).

**What is added by this report?**

CDC assessed the risk posed by this ongoing outbreak to the U.S. population during the next 3 months as low.

**What are the implications for public health practice?**

Ensuring sufficient public health resources to control the outbreak in DRC will be necessary for maintaining a low risk to the U.S. population. If cases arise in the United States, there is public health capacity to contain and control an outbreak, and CDC guidance for U.S. clinicians and public health practitioners can help prevent the potential spread.

## Abstract

On May 15, 2026, the ministries of health in the Democratic Republic of the Congo and Uganda declared outbreaks of Bundibugyo virus disease (BVD), a type of Ebola disease. In response to reports of high numbers of suspected cases and deaths in the affected countries, CDC assessed the risk posed by the BVD outbreak to the U.S. population during the next 3 months. This analysis used a standardized risk assessment approach that included epidemiologic data from the ongoing outbreak and historical data from previous Ebola outbreaks; the overall risk was determined by taking into account independent assessments of the likelihood of infection and the impact of infection. The assessment found that the overall risk to the U.S. population posed by the current BVD outbreak during the next 3 months is low, based on the extremely low likelihood of transmission, despite the high impact that potential infection could have and the resources that would be required to respond to the outbreak. Limitations to this assessment included uncertainties around the epidemiology of BVD as well as the current and future scope and geographic spread of the outbreak. CDC continues to monitor factors that could change this risk assessment.

## Introduction

Bundibugyo virus disease (BVD), a type of Ebola disease, is a severe and often fatal viral hemorrhagic fever caused by Bundibugyo virus (species *Orthoebolavirus bundibugyoense*). No vaccines or medications have been approved for BVD. As of June 2, 2026, a total of 378 confirmed BVD cases and 63 deaths have been reported in the Democratic Republic of the Congo (DRC) and Uganda (*1*). CDC assessed the potential public health implications of this BVD outbreak to the U.S. population during the next 3 months. The purpose of this risk assessment was to guide the development and implementation of U.S. preparedness efforts, including risk communication.

## Methods

CDC subject matter experts in risk assessment methodology, infectious disease modeling, global health, and Ebola disease and viral hemorrhagic fevers collaborated to develop this assessment. These experts used a standardized risk assessment approach that has been applied to previous viral hemorrhagic fever outbreaks (*2*). They considered available evidence including epidemiologic data from the ongoing BVD outbreak and historical data on Bundibugyo virus and other Ebola disease outbreaks.

Overall risk to the U.S. population was determined by independently assessing two factors: 1) the likelihood of infection and 2) the impact of infection^†^ (*2*). The likelihood of infection refers to the probability that members of the U.S. population would acquire Bundibugyo virus infection during the next 3 months; this, in turn, depends on the likelihood of exposure, infectiousness of the virus, and susceptibility of the population. The impact of infection refers to the consequences of infection in this population. Factors include the severity of disease, level of population immunity to severe disease, availability of medications and vaccines, and necessary public health response resources. Risk was assessed only for the general U.S. population; however, subpopulations that might have different assessments of likelihood are noted, based on varying risk factors. Experts then assigned a degree of confidence to the assessment, taking into account evidence quality, extent, and corroboration of information. This activity was reviewed by CDC, deemed not research, and conducted consistent with applicable federal law and CDC policy.^§^

## Results

CDC assessed the overall risk posed by the ongoing BVD outbreak to the U.S. population during the next 3 months as low. This assessment was made with moderate confidence, given the data available. This overall risk was determined based on a combination of extremely low likelihood of infection, but high impact of infection for the U.S. population, were it to occur.

### Likelihood of Infection

The likelihood of Bundibugyo virus infection for the U.S. population was assessed as extremely low. The initial reported case numbers in this outbreak are larger than initial case reports from many recent Ebola disease outbreaks, suggesting that transmission might have been ongoing for an extended period before the outbreak was recognized (*3*). Despite the large number of cases identified at the time the outbreak was reported, the current likelihood for potential spread of BVD from DRC to the United States, via travelers from DRC who might be infected, is considered very low based on modeling results that consider population movement. These modeling results suggested that the relative risk of importation to the United States compared with other locations was 1.3% (*4*). In addition, on May 18, enhanced traveler screening and entry restrictions were established to further reduce the potential for importation of BVD into the United States.

If BVD were to be introduced into the United States, based on historical observation and the known epidemiology of BVD, secondary transmission would likely be minimal. The United States has the public health capacity to rapidly implement case identification, laboratory confirmation, isolation of patients, contact tracing, and infection prevention and control measures that can contain and control an outbreak. Although BVD symptoms can appear suddenly and might be nonspecific, these public health measures are highly effective against Ebola disease, in part because the average interval between cases is long (10–16 days), and because persons are not known to be infectious before the onset of symptoms (*5*). Only 11 persons infected with Ebola disease have ever been treated in the United States; all were associated with the 2014–2016 Ebola virus disease outbreak in West Africa (*6*). Despite two instances of secondary transmission to U.S. health care workers during that outbreak, no community spread occurred in the United States. Although the likelihood of infection for the general U.S. population is low, the likelihood of infection might be higher among U.S. health care workers practicing in or who have recently returned from affected regions in DRC and Uganda based on possible exposure risks.

### Impact of Infection

The impact of infection, based on the standardized framework, was assessed as high, primarily based on the severity of the illness, lack of available medications and vaccines, and resources required to respond to the current outbreak. In the two previously identified outbreaks of BVD in Uganda in 2007 and DRC in 2012, case-fatality rates ranged from 25% to 50%. However, many of the deaths in these outbreaks occurred in locations where health resources are limited; clinical outcomes might improve with the specialized care available in the United States.

No approved vaccines or medications are currently available for BVD. A licensed vaccine and two licensed monoclonal antibody products have been used in previous outbreaks of Ebola disease caused by a different virus (species *Orthoebolavirus zairense*); whether these products are effective against BVD is unknown. While investigational medications are being evaluated, treatment for BVD is currently limited to supportive care.

Preventing spread of Bundibugyo virus requires considerable public health resources and risk communication. Public health interventions could include extensive contact tracing activities, quarantine of 21 days for persons with high-risk exposures, and stringent infection prevention and control measures for health care workers and laboratory personnel. Even very limited numbers of BVD cases in the United States might cause substantial concern among the public, possibly with some disruption of normal societal activities and to health care facilities.

### Confidence Level

Confidence in this assessment of BVD risk to the U.S. population during the next 3 months was assessed as moderate, based on availability of credible information from reliable sources, requiring minimal assumptions to be made for the analysis. The United States is prepared to respond to imported cases, and the largest previous outbreaks of Ebola disease in other countries led to very few cases within the United States. However, this assessment also recognizes uncertainties about the epidemiology of BVD, the scope and geographic spread of the outbreak, and the potential timelines for implementation of interventions.

## Discussion

CDC assessed the risk to the U.S. population posed by the current BVD outbreak as low during the next 3 months, based on the extremely low likelihood of infection, despite the high impact of infection, should it occur, and the resources required to respond to the outbreak. This assessment aligns with similar analyses conducted by other international public health organizations (*7*–*9*). Several factors could alter this assessment, including detection of any BVD cases in the United States; evidence suggesting increased transmissibility or changed clinical severity compared with previous outbreaks; or spread of the outbreak to urban, international hubs, which could increase the likelihood of importation into the United States. The emergence of additional evidence related to these factors could warrant an update to this assessment.

### Limitations

The findings in this report are subject to at least three limitations related to key uncertainties about this outbreak. First, given that only two previous BVD outbreaks have occurred, less is known about Bundibugyo virus than other types of orthoebolaviruses that cause human illness (e.g., *O. zairense*, which was responsible for the 2014–2016 Ebola virus disease outbreak in West Africa). Second, limited confirmatory diagnostic testing and challenges in contact tracing that likely resulted in undetected transmission mean that the full scope of the current outbreak is unclear, making it difficult to assess the potential geographic spread of the outbreak and the subsequent potential for spread to the United States. Nonetheless, even the largest previous outbreak of Ebola led to very few cases among U.S. persons (*6*). Finally, although DRC and neighboring countries have extensive experience responding to outbreaks of viral hemorrhagic fevers, several features of this outbreak pose challenges to assessing its future trajectory (*3*), including that the outbreak is occurring in a region with ongoing conflict and unpredictable infrastructure (*1*), and the initial large size of the outbreak combined with limited confirmatory diagnostic testing might lead to challenges in infection prevention and control efforts (*10*).

### Implications for Public Health Practice

To help ensure that the risk to the U.S. population remains low, efforts are underway to reduce the likelihood of importation to the United States and to respond quickly and effectively to any cases that might occur in the United States (*1*). Sustained public health actions are needed to slow the spread of this outbreak at its epicenter, prevent additional cases and deaths, and reduce the risk for spread to additional regions or countries (*3*). If a person in the United States is suspected to have BVD, early implementation of CDC guidance for U.S. clinicians and public health practitioners for Ebola diseases and viral hemorrhagic fevers can help prevent further transmission. Leveraging experience from past Ebola outbreaks, U.S. federal, state, tribal, local, and territorial public health, clinical, and laboratory partners will continue efforts to prevent and prepare for a possible BVD case in the United States.

## Footnotes

† Overall risk is assessed at a predefined level (extremely low, very low, low, moderate, high, and very high) with varying combinations of assessed likelihood and impact.

§ 45 C.F.R. part 46, 21 C.F.R. part 56; 42 U.S.C. Sect. 241(d); 5 U.S.C. Sect. 552a; 44 U.S.C. Sect. 3501 et seq.
